# Evaluation of Viability and Adhesion of Human Gingival Fibroblast and the Adhesion of Oral Microflora on Thermally Aged Zirconia and BioHPP Abutment Surfaces: An In Vitro Study

**DOI:** 10.1155/ijod/1553672

**Published:** 2025-06-04

**Authors:** Ishita Singh, Umesh Y. Pai, B. Thilak Shetty, Shobha J. Rodrigues, Sharon Saldanha, M. Mahesh, K. Shama Prasada, Vignesh Kamath, Prashant Bajantri, Sandipan Mukherjee, Ann Sales

**Affiliations:** ^1^Private Practice, Department of Prosthodontics, Smile Factory Dental Clinic, Calangute, Goa, India; ^2^Department of Prosthodontics and Crown and Bridge, Manipal College of Dental Sciences Mangalore, Manipal Academy of Higher Education, Manipal 576104, Karnataka, India; ^3^Department of Cell and Molecular Biology, Manipal School of Life Sciences, Manipal Academy of Higher Education, Manipal 576104, Karnataka, India

**Keywords:** BioHPP, human gingival fibroblasts, implant abutment, LTD, MTT assay, ZrO_2_

## Abstract

**Background:** The objective of the study is to evaluate the effect of thermal aging of zirconia and BioHPP abutment surfaces on the viability and adhesion of human gingival fibroblasts (HGFs) and multispecies biofilm formation of *Escherichia coli* (*E. coli*) and *Streptococcus mutans* (*S. mutans*).

**Materials and Method:** The study utilized circular disks of zirconia oxide (ZrO_2_) and BioHPP measuring 8 × 2 mm. The surface roughness was evaluated for both nonaged and aged specimens. The HGF viability was evaluated by using a 3-(4,5-dimethylthiazol-2-yl)-2,5-diphenyltetrazolium bromide (MTT) assay, adhesion using scanning electron microscopy (SEM). Multispecies biofilms were analyzed quantitatively by using a software ImageJ and qualitatively by SEM. One-way ANOVA was used paired with post hoc Tukey test for the statistical analysis.

**Results:** The surface roughness of BioHPP was significantly more than that of zirconia, and aged zirconia had significantly more surface roughness than nonaged zirconia. In terms of cell viability, zirconia showed higher viability of HGF compared to BioHPP where aged specimens had lesser cell viability than nonaged specimens. BioHPP had a higher adhesion rate of *S. mutans* and *E. coli* in comparison to zirconia, and aged specimens had a higher adhesion rate compared to nonaged specimens.

**Conclusion:** Surface roughness affects the bioactivity of the cells. Therefore, for the longevity of implants, a smoother surface is preferred. Even though zirconia showed better results in terms of HGF viability and microbial adhesion in comparison to BioHPP, the smaller differences between the two show that BioHPP can be used as an alternate to zirconia in esthetically driven cases. But both the materials show that aging affects the physicochemical properties and bioactivity of the surrounding cells.

## 1. Introduction

Over the last 20 years, the field of implant dentistry has markedly evolved to provide long-term successful and predictable treatment outcomes with many biological and mechanical advantages over conventional prostheses. Due to the increased concerns of patients regarding esthetics as well as biocompatibility concerns with dental alloys, ceramics have gained popularity. In the past few years, research in metal-free restorations has led to the development of innovative ceramic materials that have optical properties which are excellent and mechanical properties better than previous dental ceramics [1].

The use of zirconia has increased in common dental practice for the fabrication of crown and bridge restorations and implant abutments, particularly in esthetic zones. Zirconia gives a very natural, healthy, and attractive appearance when in contact with the marginal gingiva or peri-implant mucosa, in both tooth-supported and implant-supported restorations resulting in better optical properties, very low plaque retention, and proven ability to induce epithelial attachment on its surface. With increasing clinical and research experience, and further development and modification of implant surfaces, the scientific focus has shifted from the investigation of osseointegration to investigating peri-implant soft tissue response to various restorative materials currently in use [2].

The low-temperature degradation (LTD) which is caused by aging phenomena during in situ clinical use is the main disadvantage of abutments made of zirconia [3]. LTD or aging results in the transformation of a metastable tetragonal structure (*t*) to a stable monoclinic structure (*m*) in yttrium-stabilized tetragonal zirconia (Y-TZP) [4]. The only zirconia to comply with ISO standard 13356:2008 is yttria-stabilized zirconia (ZrO_2_–Y_2_O_3_), which states that after LTD done for 5 h at 134°C and 2 bar pressure and that less than 25% should constitute the monoclinic phase. Chevalier et al. [5] in his study stated that 1 h of autoclave at 134°C and 2 bar pressure causes a transformation of tetragonal to monoclinic (*t* to *m*) transformation which is equivalent to 3–4 years of the material in clinical services.

It is important to be aware of this transition from *t* to *m* and its consequences on the long-term biological and mechanical properties in clinical practise if zirconia is to be used as a dependable material for prosthetic restorations. When external forces cause this transformation, it causes an expansion and change of shape within individual grains, which absorbs energy and enhance damage resistance.

BioHPP is a ceramic reinforced high-performance polymer that was created and optimized specifically for intraoral use. It has a wide range of properties, including a high coefficient of elasticity (comparable to bone), water solubility being low, high wear resistance, increased biocompatibility, processing ease, and improved esthetics. The key advantage for using BioHPP is that it has lower Young's (elastic) moduli (3–4 GPa), which is similar to that of human bone [6].

Given that rough surfaces have been linked to decreased epithelial downgrowth and a more coronally positioned gingival tissue adaptation, roughness below 0.2 mm may be the most efficient strategy to create a barrier of defense surrounding the abutments. Due to their supra and subgingival locations, abutment appears to be of critical importance for biofilm production. As a result, the materials utilized to make implant abutments should prevent bacterial growth on their surface [7].

Prior studies indicated in vivo protein adsorption and adhesion of bacteria can be influenced by threshold a 0.2-µm surface roughness when it comes to the impact of surface roughness on biofilm formation [8]. The gingival fibroblast biological behavior studies in term of dental materials are important in the prosthodontic field because these cells play a role in the oral soft tissue response to biomaterials. In particular, gingival fibroblasts participate in the integration process with ceramic materials, determining whether or not biomaterials can be integrated with them.

Thus, the purpose of this study is to evaluate the effect of in vitro aging on the physicochemical properties of zirconia oxide (ZrO_2_) and BioHPP abutment materials. The effects of aging on viability and adhesion of human gingival fibroblasts (HGFs) and the multispecies biofilm formation of *Escherichia coli* (*E. coli*) and *Streptococcus mutans* (*S. mutans*) were investigated. The null hypothesis tested will be that aging of the abutment materials, ZrO_2_ and BioHPP, does not influence the viability and adhesion of HGF nor the multispecies biofilm formation of *E. coli* and *S. mutans*.

## 2. Materials and Method

The sample size was calculated assuming a 95% level of confidence and 80% power for the study based on previous studies. A sample size of 3 per group gave a power of 99% to detect a clinically significant difference of 8.3 units with an average standard deviation of differences of 2.5. Depending on the type of abutment materials used, the groups were divided as follows: (1) Group 1, nonaged zirconia disks; (2) Group 2, aged zirconia disks; (3) Group 3, nonaged BioHPP disks; and (4) Group 4, aged BioHPP disks.

**Figure 1:**
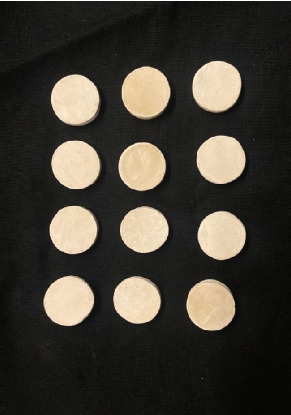
BioHPP disks.

**Figure 2:**
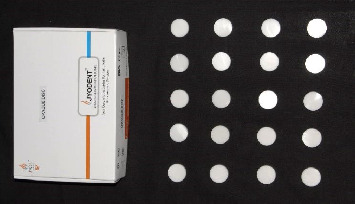
Zirconia disks.

A total of 60 samples will be considered in this study. Thirty BioHPP disks (Figure 1) and 30 opaque zirconia disks (Figure 2) measuring 8 mm in diameter and 2 mm in height will be prepared. Zirconia disks of diameter 8 mm and thickness 2 mm were obtained from Jyothi Ceramics (Mumbai, India). The disks were milled. BioHPP disks of diameter 8 mm and thickness 2 mm were obtained from Bredent Hyderabad. The disks were milled to the size. They were then smoothened and polished. The disks were then subjected to sandpapering using 800 grit, 1200 grit, 2000 grit, and 4000 grit silicon carbide paper. For surface roughness evaluation, X-ray diffraction (XRD) was performed using an XRD device at Central Instrumentation Facility, Manipal. The disks were then cleaned by immersing them in acetone for 20 min and then transferring to distilled water after which ultrasonic cleaning for 20 min and a final thorough wash with distilled water.

**Figure 3:**
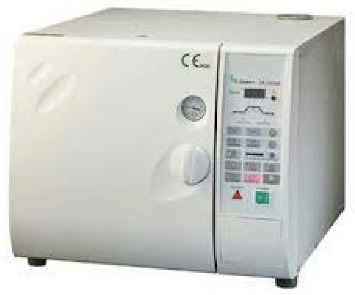
Autoclave (Sturdy SA 230-MA).

**Figure 4:**
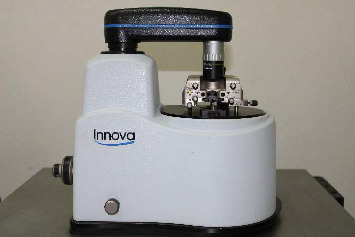
Atomic force microscopy.

To simulate LTD, half of the disks were placed in an autoclave (Figure 3) at 134°C for 10 h and a pressure of 2 bar [7]. On completion of the aging process, the disks were tested for surface roughness through atomic force microscopy (AFM) (Figure 4).

The disks were placed in a 12-well plate with one disk per plate, and the cells were cultivated at a density of 20,000 cells per well. The cells were cultured with 1 mL of media and allowed to grow and adhere to confluence over 48 h. 3-(4,5-Dimethylthiazol-2-yl)-2,5-diphenyltetrazolium bromide (MTT) assay was performed to check for HGF cell culture viability. The test was performed in duplicate. The disks were incubated in the media for 4 h after which the media were discarded. HGF viability was evaluated after 48 h on the disks by MTT assay. The plate was incubated in DMSO solution for 30 min at 37°C and then read at 570 nm. The viable cells were able to reduce MTT into a colored formazan product that is purple in color [1, 6, 7].

**Figure 5:**
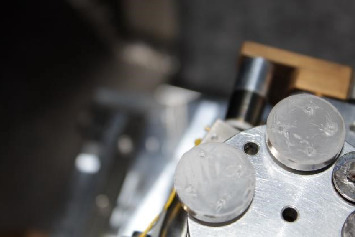
Gold sputtering of samples for SEM analysis [2].

Scanning electron microscopy (SEM) was performed using Carl ZEISS EVO 10 SEM to check for adhesion of HGF onto the aged and nonaged BioHPP and zirconia disks (one disk from per group). The disks were gold sputtered prior to the scanning (Figure 5). The images recorded were at 7000× and 10,000× magnification.

**Figure 6:**
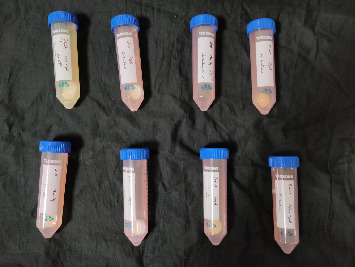
Samples placed in *S. mutans* and *E. coli* media.

**Figure 7:**
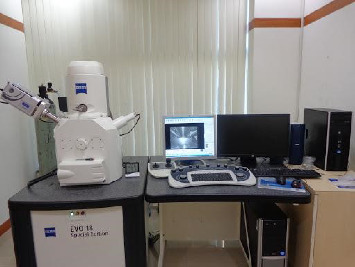
Scanning electron microscope.

**Figure 8:**
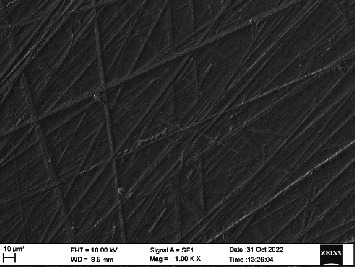
SEM image of ZrO_2_ NA (1000×).

**Figure 9:**
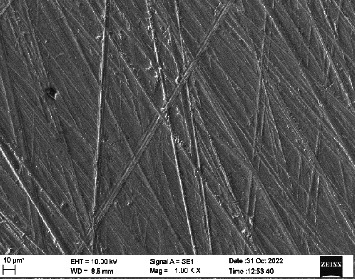
SEM image of ZrO_2_ A (1000×).

**Figure 10:**
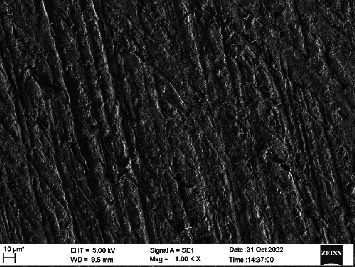
SEM image of BioHPP NA (1000×).

**Figure 11:**
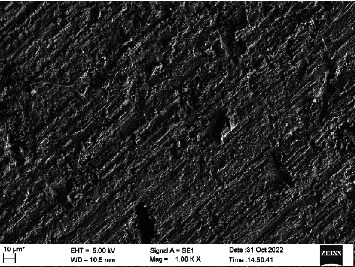
SEM image of BioHPP A (1000×).

*S. mutans* and *E. coli* were cultured using brain heart infusion for 48 h. The separate cultures were divided into eight tubes, four for *S. mutans* and four for *E. coli* for the four groups. Three disks per group were placed in their designated test tube. The disks stayed in the medium for 48 h which allowed the microflora to grow onto the disks (Figure 6). One disk per group was kept as control. SEM was performed using Carl ZEISS EVO 18 SEM (Figure 7) to check for adhesion of *S. mutans* and *E. coli* onto the aged and nonaged BioHPP and zirconia disks. The images recorded were at 7000× and 10,000× magnification (Figures 8–11).

## 3. Results

**Figure 12:**
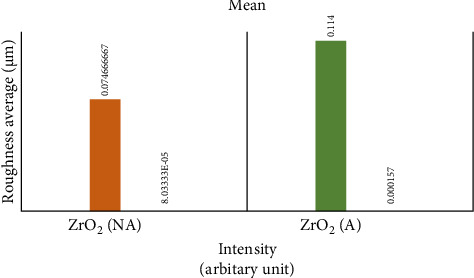
Mean surface roughness (µ) between nonaged and aged ZrO_2_.

**Figure 13:**
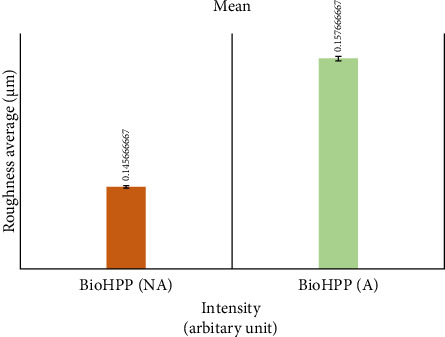
Mean surface roughness (µ) between nonaged and aged BioHPP.

The roughness data (Tables 1 and 2) showed that there was a significant difference between the roughness values of various groups with *p* < 0.000. There was a difference in the mean surface roughness before and after the aging process of both ZrO_2_ and BioHPP. The highest mean recorded was of aged BioHPP followed by nonaged BioHPP, aged ZrO_2_, and the lowest of nonaged ZrO_2_. An increase in the surface roughness was seen after aging for both ZrO_2_ and BioHPP groups as shown in Figures 12 and 13.

**Figure 14:**
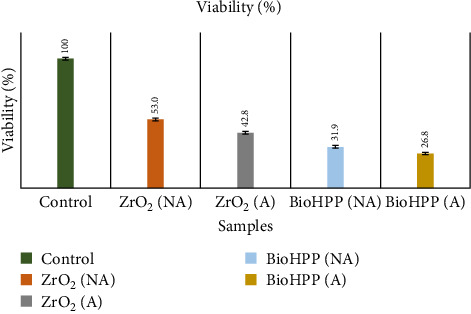
Human gingival fibroblast (HGF) cell viability between nonaged and aged ZrO_2_ and BioHPP.

The cell viability (Table 3) results show that nonaged ZrO_2_ showed the highest viability followed by aged ZrO_2_ and nonaged BioHPP, and the least viability was seen in aged BioHPP (Figure 14). One-way ANOVA (Table 4) showed there was a significant difference between the groups in terms of viability with *p* < 0.00. Post hoc Tukey test (Table 5) showed that there was a significant difference between aged ZrO_2_ and nonaged ZrO_2_ (*p* < 0.0002), between aged ZrO_2_ and nonaged BioHPP (*p* < 0.0001), between aged ZrO_2_ and aged BioHPP (*p* < 0.000), between nonaged ZrO_2_ and aged BioHPP (*p* < 0.005), and between nonaged ZrO_2_ and nonaged BioHPP (*p* < 0.000). There was no significant difference between nonaged and aged BioHPP.

**Figure 15:**
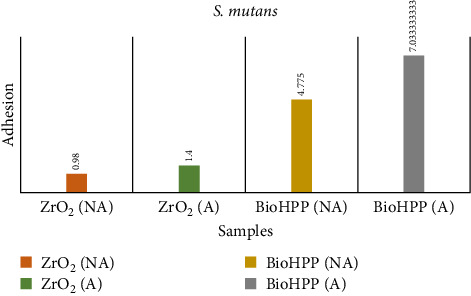
Adhesion of *S. mutans* on nonaged and aged ZrO_2_ and BioHPP.

**Figure 16:**
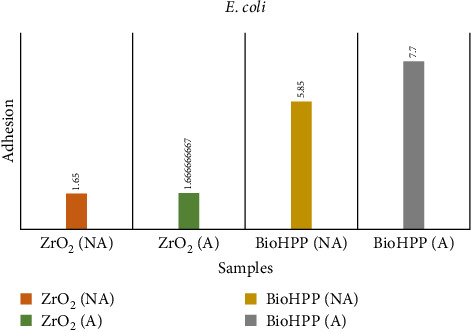
Adhesion of *E. coli* on nonaged and aged ZrO_2_ and BioHPP.

The results for adhesion of *S. mutans* and *E. coli* (Table 6) showed that aged BioHPP recorded the highest count of adhesion followed by nonaged BioHPP, aged zirconia, and the least with nonaged zirconia (Figures 15 and 16).

The one-way ANOVA analysis shows that there is significant difference between the four groups of zirconia and BioHPP with and without aging. Post hoc Bonferroni's test shows that the significant difference is between aged ZrO_2_ and aged BioHPP (*p* < 0.00028), between nonaged ZrO_2_ and nonaged BioHPP (0.0097), and between nonaged ZrO_2_ and aged BioHPP (*p* < 0.00027) (Table 7). Post hoc Bonferroni's test for *E. coli* showed that the significant difference was between aged ZrO_2_ and nonaged BioHPP (*p* < 0.0068), between aged ZrO_2_ and aged BioHPP (*p* < 0.000293), between nonaged ZrO_2_ and nonaged BioHPP (*p* < 0.00479), and between aged ZrO_2_ and aged BioHPP (*p* < 0.00022) (Table 8).

## 4. Discussion

In an implant, abutment is the only part that is in contact with the implant screw, the bone, and the soft tissue. Therefore, it is of the utmost importance for the implant abutment to be made of a biocompatible material, to ensure the longevity of implants. Titanium has been the gold standard material in use. However, in esthetically demanding patients especially in the anterior region, the metallic hue of the titanium seems unpleasant, thereby making zirconia the material of choice in such cases.

BioHPP, ceramic-reinforced PEEK manufactured by Bredent, UK, is another such material for use in esthetically demanding areas. The promising property of BioHPP apart from the color is its elastic modulus similar to bone, unlike zirconia and titanium, whose values are much higher. However, very limited studies have been done regarding the biochemical properties of BioHPP.

In the present study, a commonly used abutment material was compared to a newly introduced material BioHPP for comparison of their physicochemical and biological properties after hydrothermal aging. Hydrothermal aging replicates the clinical years in an in vitro study. Chevalier et al. [5] in his study stated that 1 h of autoclave at 134°C and 2 bar pressure causes a transformation of tetragonal to monoclinic (*t* to *m*) transformation which is equivalent to 3–4 years of the material in clinical services. The physicochemical properties of ZrO_2_ and BioHPP abutments were shown to be affected by the aging phenomenon. There was also a difference seen in the viability and adhesion of HGF and adhesion of *S. mutans* and *E. coli* on the ZrO_2_ and BioHPP abutment surfaces before and after aging. Therefore, the null hypothesis was rejected that aging of the abutment materials ZrO_2_ and BioHPP did not influence the viability and adhesion of HGF or the multispecies biofilm formation of *S. mutans* and *E. coli*

The surface roughness of ZrO_2_ had a significant difference before and after aging with the values increasing after aging (Table 1). There was no significant difference in the surface roughness values of BioHPP abutments before and after aging. An increase in the average roughness values can be seen in aged zirconia implant abutments (Table 2). Similar results were seen in a study conducted by Peampring et al. [9], where he observed a significant increase in the surface roughness of ZrO_2_ abutments after hydrothermal aging. In another study done by Pandoleon et al. [8], a significant increase in roughness was observed after 5 h of aging of zirconia. In a study done by Rigolin et al. [7], no significant differences were observed in the roughness values of zirconia before and after aging.

The surface roughness of BioHPP abutments was recorded to be more than that of ZrO_2_ abutments. Similar results were observed in a study done by Khalifa et al., although PEEK was used instead of BioHPP. In a study done by Giudice et al. [10], where he compared surface roughness values for Zirconia and BioHPP, opposing results were seen where the surface roughness values of BioHPP were significantly less than that of zirconia surfaces.

**Figure 17:**
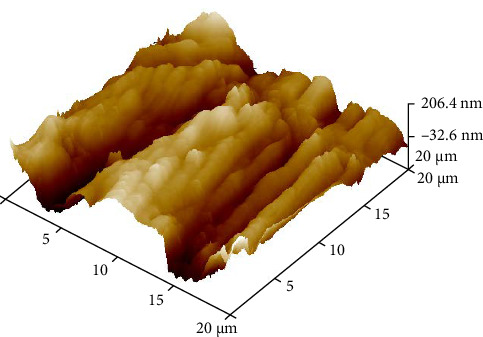
AFM analysis of ZrO_2_ NA.

**Figure 18:**
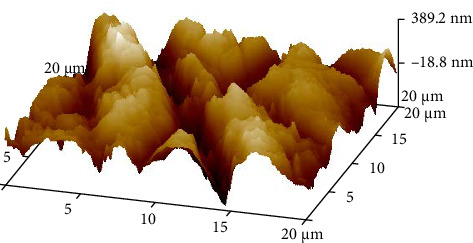
AFM analysis of ZrO_2_ A.

**Figure 19:**
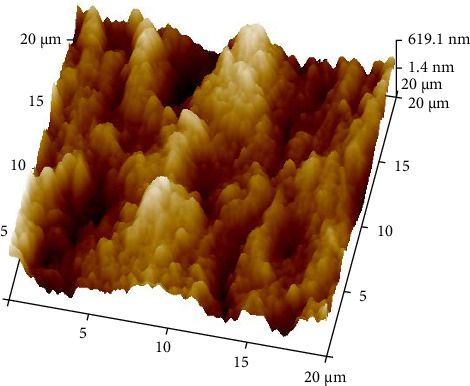
AFM analysis of BioHPP NA.

**Figure 20:**
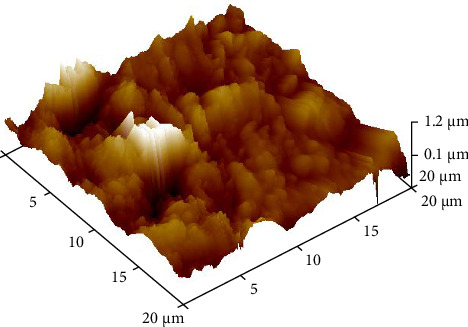
AFM analysis of BioHPP A.

The AFM analysis images showed that the peaks were lower for nonaged ZrO_2_ and nonaged BioHPP compared to aged ZrO_2_ aged BioHPP, respectively, thereby indicating that the surface roughness was more for the aged specimens compared to nonaged specimens. The number of peaks and valleys is more for BioHPP compared to ZrO_2_, indicating that BioHPP has a surface roughness higher than ZrO_2_ (Figures 17–20). In the study done by Rigolin et al. [7], AFM and XRD was used to study the surface roughness of the specimens. In the study done by Ramenzoni et al. [11], a surface profilometer was used to evaluate the surface roughness of the materials. In a study done by Pandoleon et al. [8], an optical profilometer was used to ascertain the surface roughness.

However, in the present study, all the values recorded were under 0.2 µm. According to some studies, 0.2-µm roughness value is the acceptable threshold value required to maintain the seal between the epithelial cells and the surface [12, 13], which also prevents the immigration of bacteria and hence the stability of the soft tissue.

Although BioHPP recorded higher values than zirconia, the difference was small, and these values were under 0.2 µm, and no polishing protocol was followed. A custom tailored polishing protocol for BioHPP may give better results.

HGFs are one of the main cell populations in the peri-implant soft tissue, apart from human gingival keratinocytes and osteoblasts. HGFs are the major cellular components of the peri-implant connective tissue (CT), secreting collagen fibers and laying down the extracellular matrix (ECM). They are further responsible for gingival wound healing, repair, regeneration, and adherence of peri-implant CT to the implant abutment surface. A good soft tissue seal is necessary for the implant survival. A good seal not only ensures a healthy implant junctional epithelium but also prevents the entry of bacteria. The bioactivity, that is, viability, proliferation, and adhesion of the cells at the implant gingival interface, may impact the peri-implant soft tissue seal [14].

In the present study, HGF cell population showed the highest viability for nonaged zirconia abutment surfaces and least for aged BioHPP abutment surfaces. A significant difference was observed between aged and nonaged zirconia abutments, with aged having lesser viability compared to nonaged. This signifies that the aging process did affect the viability of the cells for both zirconia and BioHPP abutment surfaces (Tables 3–5).

**Figure 21:**
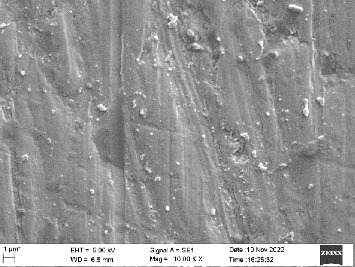
SEM image of HGF adhered on ZrO_2_ NA (10,000×).

**Figure 22:**
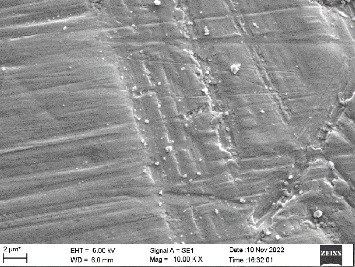
SEM image of HGF adhered on ZrO_2_ A (10,000×).

**Figure 23:**
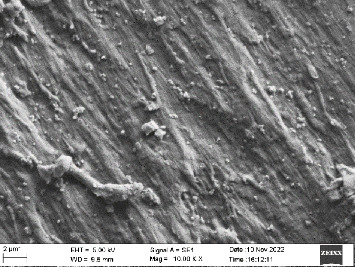
SEM image of HGF adhered on BioHPP NA (10,000×).

**Figure 24:**
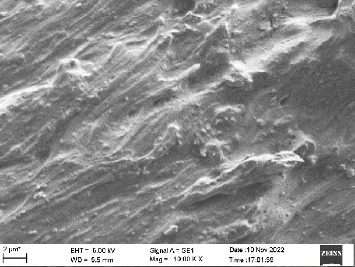
SEM image of HGF adhered on BioHPP A (10,000×).

The SEM images of HGF show that the cells appeared mostly like single isolated elements and round shaped. The adherence of the cells as per the SEM images is more for ZrO_2_ compared to BioHPP and more for nonaged specimens compared to aged specimens (Figures 21–24).

The results obtained are the same as in the study done by Rozeik et al. [15], where he observed that the cell viability for ZrO_2_ specimens were more as compared to PEEK specimens. In another study done by Rigolin et al. [7], no significant difference was observed in the cell viability among the aged and nonaged zirconia samples. In that same study, no significant difference was observed in the surface roughness values as well. In a study done by Pandoleon et al. [8], he observed that the viability of HGF decreased as the number of hours of aging increased. In a study done by Osman et al. [14], he observed that the viability of HGF cells was more for m-PEEK (modified with ceramic and glass fillers) as compared with ZrO_2_. In the same study, the surface roughness for m-PEEK was lesser compared to ZrO_2_. It can be attributed that the surface roughness has an effect on the viability and adhesion of cells on the abutment surfaces with smoother surfaces having more viability and adhesion. The difference in the results could be due to variations in cell culture times and types of cells and assays used.

Zirconia is a chemically inert material, therefore ensuring the higher viability percentage. The lower viability of cells on BioHPP could be as described in a study, in which PEEK inhibits the processing of cellular mRNA, resulting in a lower rate of cell proliferation on its surface. PEEK is further hydrophobic in nature, impacting the spreading and attachment of cells on its surface.

The interplay between the implant abutment and peri-implant tissue governs the long-term success of the dental implant. Therefore, it is necessary for a good seal, which is governed by the bioactivity of the HGF and other cells [15].

In terms of the microflora adhesion of *S. mutans* and *E. coli* to the abutment surfaces, a higher percentage of *E. coli* was seen to adhere to both zirconia and BioHPP abutment surfaces. The highest adhesion values observed for *S. mutans* were for aged BioHPP and the least for nonaged zirconia. A significant difference was seen among the groups. Similarly for *E. coli*, aged BioHPP had the highest values, and nonaged Zirconia had the least values. These results can be attributed to the surface roughness values, where it was seen that aged BioHPP had the maximum and nonaged zirconia had the least surface roughness values (Tables 6–8).

**Figure 25:**
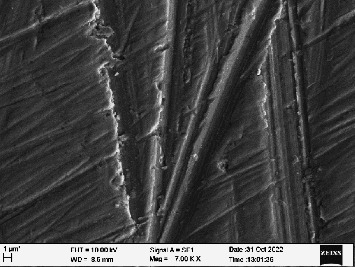
SEM image *S. mutans* adhered on ZrO_2_ NA (7000×).

**Figure 26:**
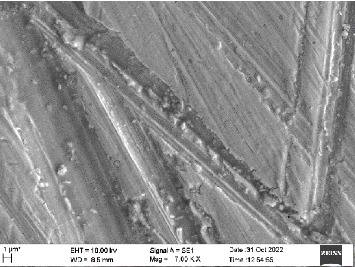
SEM image *S. mutans* adhered on ZrO_2_ A (7000×).

**Figure 27:**
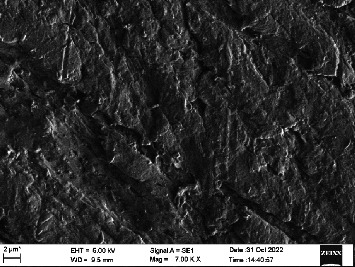
SEM image *S. mutans* adhered on BioHPP NA (7000×).

**Figure 28:**
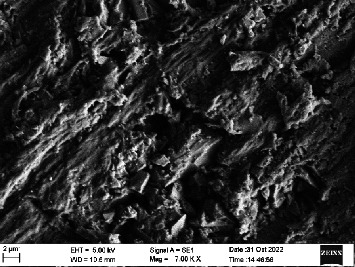
SEM image *S. mutans* adhered on BioHPP A (7000×).

**Figure 29:**
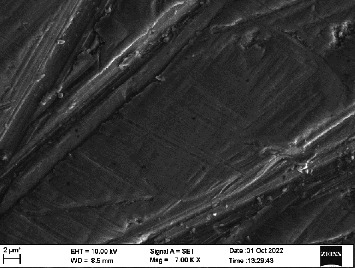
SEM image *E. coli* adhered on ZrO_2_ NA (7000×).

**Figure 30:**
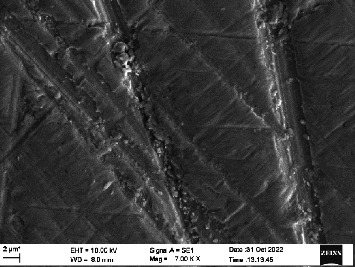
SEM image *E. coli* adhered on ZrO_2_ A (7000×).

**Figure 31:**
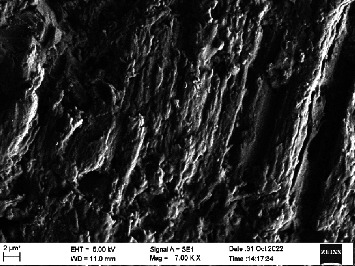
SEM image *E. coli* adhered on BioHPP NA (7000×).

**Figure 32:**
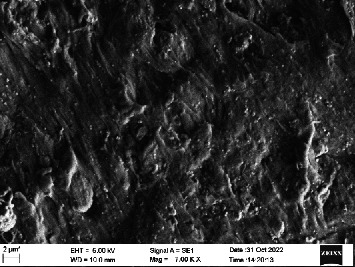
SEM image *E. coli* adhered on BioHPP A (7000×).

There was a significant difference seen in the adherence values between the aged and nonaged groups, with the aged groups having significantly higher values of adherence in both zirconia and BioHPP. The SEM images exhibited BioHPP showed a higher adherence to the microflora compared to ZrO_2_ with aged specimens having a higher adherence to nonaged specimens (Figures 25–32).

In the study done by Rigolin et al. [7], a higher percentage of microbial adhesion was seen on aged zirconia compared to nonaged zirconia, similar to the present study. According to the study done by Hahnel et al. [16], PEEK showed a lower percentage of microbial adhesion compared to zirconia abutment surfaces along with lower surface roughness observed for PEEK compared to zirconia.

Koutouzis' controlled clinical research (randomized controlled trial [RCT]) shows that there are no appreciable differences between PEEK and titanium superstructures in terms of bone resorption and soft tissue inflammation [17]. It has also been demonstrated in studies that the value of the oral microflora adhering to PEEK is comparable to that of Ti and ZrO_2_ based ceramic abutments [17–19].

Quirynen and Bollen [20] concluded in his study that rougher surfaces lead to an increased microbial adhesion and hence a higher plaque accumulation. Hahnel et al. [16] reported that rougher surfaces of implant surfaces may have an effect on the microbial adherence, which might suggest that rougher surfaces lead to a higher percentage of microbial adhesion. Therefore, smoother surfaces are required for better long-term survival of the implants.

More studies are required to assess the biological behavior of both the cells and microorganisms on the implant abutment surfaces, especially for BioHPP. In the current study, the aging process had a significant effect on all properties; thereby, further studies should be done utilizing different methods of aging. Understanding the correlation between the physicochemical and biological properties of the abutment surface is necessary to establish a good peri-implant soft tissue health which in turn is essential for the longevity of the implants.

More physical properties like surface tension can be tested to evaluate and compare the cell adherence of HGFs. There are cell lines other than HGFs, that is, human gingival keratinocytes and human osteoblasts. Chemical properties can be tested with the cell lines for the materials. Intraoral conditions with saliva and plaque biofilm could not be replicated in this in vitro study.

## 5. Conclusion

• The surface roughness of BioHPP was significantly more than that of zirconia, with aged zirconia exhibiting significantly more surface roughness than nonaged zirconia and no significant difference between nonaged and aged BioHPP.
• Zirconia showed higher viability of HGF compared to BioHPP and aged specimens of both materials had lesser cell viability than nonaged specimens.
• BioHPP had a higher adhesion rate of *S. mutans* and *E. coli* in comparison to zirconia, and aged specimens had a higher adhesion rate compared to nonaged specimens.

## Figures and Tables

**Table 1:** Mean surface roughness (µ) between nonaged and aged ZrO_2_ and BioHPP.

	ZrO_2_ NA	ZrO_2_ A	BioHPP NA	BioHPP A
Mean (Sa)	0.07466667	0.114	0.145667	0.157667

**Table 2:** Surface roughness (µ) comparison between nonaged and aged ZrO_2_ and BioHPP.

Groups	Count	Sum	Average	Variance	*F*	*p*-Value
ZrO_2_ NA	2	0.342	0.07466667	0.00008	29.46643	0.000113
ZrO_2_ A	2	0.224	0.114	0.000157		
BioHPP NA	2	0.437	0.145667	0.000121		
BioHPP A	2	0.473	0.157667	0.000202		

**Table 3:** Viability of human gingival fibroblast on nonaged and aged zirconia and BioHPP.

Samples	Reading 1	Reading 2	Average	Viability (%)	Viability (%)	Viability (%)	Standard deviation
Control	0.7385	0.7478	0.74315	99.37429	100.6257	100	0.884894
ZrO_2_ (NA)	0.384	0.4039	—	51.67194	54.34973	53.01083	1.893484
ZrO_2_ (A)	0.3013	0.3344	—	40.54363	44.99765	42.77064	3.149463
BioHPP (NA)	0.2324	0.2424	—	31.27229	32.61791	31.9451	0.951499
BioHPP (A)	0.2002	0.1984	—	26.93938	26.69717	26.81827	0.17127

**Table 4:** One-way ANOVA comparing zirconia and BioHPP specimens.

Samples	Viability (%)	Standard deviation	*F*	*p*-Value
Control	100	0.9	842.6642	0
ZrO_2_ (NA)	53.0	1.9		
ZrO_2_ (A)	42.8	3.1		
BioHPP (NA)	31.9	1.0		
BioHPP (A)	26.8	0.2		

**Table 5:** Post hoc Tukey test.

Groups	Diff	CI	*p*
Control vs. BioHPP (A)	−73.1817	−77.8704 to −68.4931	0.9439
Control vs. BioHPP (NA)	−68.0549	−72.7435 to −63.3663	0.9303
Control vs. ZrO_2_ (NA)	−46.9892	−51.6778 to −42.3005	0.6709
Control vs. ZrO_2_ (A)	−57.2294	−61.9180 to −52.5407	0.8590
BioHPP (A) vs. BioHPP (NA)	5.1268	0.4328	0.0310
BioHPP (A) vs. ZrO_2_ (NA)	26.1926	21.5039 to 30.8812	0.0052
BioHPP (A) vs. ZrO_2_ (A)	15.9524	11.2637 to 20.6410	0.0000
BioHPP (NA) vs. ZrO_2_ (NA)	21.0657	16.3771 to 25.7544	0.0000
BioHPP (NA) vs. ZrO_2_ (A)	10.8255	6.1369 to 15.5142	0.0001
ZrO_2_ (NA) vs. ZrO_2_ (A):	−10.2402	−14.9288 to −5.5516	0.0002

**Table 6:** Zirconia (ZrO_2_) and BioHPP.

Zirconia (ZrO_2_)				BioHPP			
*S. mutans*		*E. coli*		*S. mutans*		*E. coli*	
Nonaged	Aged	Nonaged	Aged	Nonaged	Aged	Nonaged	Aged
No. of bacteria/100 µM^2^		No. of bacteria/100 µM^2^		No. of bacteria/100 µM^2^		No. of bacteria/100 µM^2^	
2.8	1.7	0.4	0.8	7.5	6.5	5	7.2
0.3	0.4	2.2	3.1	6.3	5.8	7.6	8.5
0.4	2.3	3.4	0.6	1.8	8.8	4.1	6.9
0.9	0.5	0.3	1.3	3.5	—	—	—
0.5	2.1	0.7	—				
0.96	1.4	1.4	1.4	4.7	7	5.6	7.5

**Table 7:** One-way ANOVA and post hoc Bonferroni's test to compare four groups: *S. mutans*.

Groups	Count	Sum	Average	Variance	*F*	*p*-Value
ZrO_2_ NA	5	4.9	0.98	1.087	12.52696	0.000391
ZrO_2_ A	5	7	1.4	0.8		
BioHPP NA	4	19.1	4.775	6.7425		
BioHPP A	3	21.1	7.033333	2.463333		

Post hoc Bonferroni's test						
Zirconia *S. mutans* nonaged vs. zirconia *S. mutans* aged						0.256746
Zirconia *S. mutans* aged vs. BioHPP *S. mutans* nonaged						0.014249
Zirconia *S. mutans* aged vs. BioHPP *S. mutans* aged						0.000284
Zirconia *S. mutans* nonaged vs. BioHPP *S. mutans* nonaged						0.009703
Zirconia *S. mutans* nonaged vs. BioHPP *S. mutans* aged						0.000276
BioHPP *S. mutans* nonaged vs. BioHPP *S. mutans* aged						0.122283

**Table 8:** One-way ANOVA and post hoc Bonferroni's test to compare four groups: *E. coli*.

Groups	Count	Sum	Average	Variance	*F*	*p*-Value
ZrO_2_ NA	4	6.6	1.65	2.03	9.722835	0.006842
ZrO_2_ A	3	5	1.666667	1.663333		
BioHPP NA	2	11.7	5.85	6.125		
BioHPP A	2	15.4	7.7	1.28		

Post hoc Bonferroni's test						
Zirconia *E. coli* nonaged vs. zirconia *E. coli* aged						0.477359
Zirconia *E. coli* aged vs. BioHPP *E. coli* nonaged						0.006856
Zirconia *E. coli* aged vs. BioHPP *E. coli* aged						0.000293
Zirconia *E. coli* nonaged vs. BioHPP *E. coli* nonaged						0.004799
Zirconia *E. coli* nonaged vs. BioHPP *E. coli* aged						0.000222
BioHPP *E. coli* nonaged vs. BioHPP *E. coli* aged						0.082415

## Data Availability

All the data will be available with the first and corresponding author.
